# The Phosphoinositide-Binding Protein ZF21 Regulates ECM Degradation by Invadopodia

**DOI:** 10.1371/journal.pone.0050825

**Published:** 2013-01-31

**Authors:** Daisuke Hoshino, Makoto Nagano, Anri Saitoh, Naohiko Koshikawa, Takashi Suzuki, Motoharu Seiki

**Affiliations:** 1 Division of Cancer Cell Research, Institute of Medical Science, The University of Tokyo, Tokyo, Japan; 2 Department of Analytical Chemistry, Faculty of Pharmaceutical Sciences, Setsunan University, Hirakata, Osaka, Japan; 3 Division of Mathematical Science, Graduate School of Engineering Science, Osaka University, Toyonaka, Osaka, Japan; Stony Brook University, United States of America

## Abstract

During the process of tumor invasion, cells require footholds on extracellular matrices (ECM) that are created by forming focal adhesions (FAs) using integrins. On the other hand, cells must degrade the ECM barrier using extracellular proteases including MMPs in the direction of cell movement. Degradation occurs at the leading edges or invadopodia of cells, which are enriched in proteases and adhesion molecules. Recently, we showed that the phosphoinositide-binding protein ZF21 regulates FA disassembly. ZF21 increased cell migration by promoting the turnover of FAs. In addition, ZF21 promotes experimental tumor metastasis to lung in mice and its depletion suppresses it. However, it is not known whether ZF21 regulates cancer cell invasion in addition to its activity on FAs. In this study, we demonstrate that ZF21 also regulates invasion of tumor cells, whereas it does not affect the overall production of MMP-2, MMP-9, and MT1-MMP by the cells. Also, we observe that the ECM-degrading activity specifically at the invadopodia is severely abrogated. In the ZF21 depleted cells MT1-MMP cannot accumulate to the invadopodia and thereby cannot contribute to the ECM degradation. Thus, this study demonstrates that ZF21 is a key player regulating multiple aspects of cancer cell migration and invasion. Possible mechanisms regulating ECM degradation at the invadopodia are discussed.

## Introduction

The metastatic spread of cancer cells is a major killer of patients and occurs as a consequence of a complex interaction between cancer cells and host tissues [1], [2]. Signals that stimulate migration and invasion of cancer cells contribute to metastasis and such metastatic cells frequently acquire autonomous mechanisms to stimulate migration and invasion [3]. Cellular migration requires dynamic regulation of the actin cytoskeleton involving cell adhesion structures that interact with the extracellular matrix (ECM) outside [4]. Such structures include focal adhesions (FAs), which are observed on cells cultured onto an ECM layer [5]. FAs comprise ECM receptor integrins, scaffold proteins, and signal molecules [6]. Binding of integrins to components of the ECM causes the former to cluster. The clustering leads to recruitment of scaffold and signaling molecules to the cytoplasmic tails of the integrins, where they mediate bidirectional signals [7]. FAs physically link the ECM structure to the actin cytoskeleton and thereby enable generation of cellular forces necessary for migration and maintenance of cellular morphology [8]. The continuous formation and disassembly of FAs is characteristic of migrating cells. In contrast, a greater number of stable FAs is characteristic of stably adhered cells on ECM.

Metastatic cancer cells are usually highly mobile and use multiple ECM-degrading proteases including MMPs to enable invasion [9]. Pericellular proteolysis coupled with migration promotes invasion of cells into the surrounding ECM. A membrane-anchored MMP, MT1-MMP, plays a central role in pericellular proteolysis of the ECM and acts as a potent proinvasive MMP [10]. These ECM-degrading enzymes including MT1-MMP mostly localize to the leading edges of invading cells [11], [12]. In some types of cell, this invasion edge forms a membrane protrusion called an “invadopodium” where cell adhesion molecules, actin, its regulators, and proteases are assembled [13]. Thus, FAs and invadopodia are characteristic cellular structures of the cell-ECM interaction, both of which are important for cancer cell invasion. These structures share some common components, such as cell adhesion molecules and regulators of the actin cytoskeleton, although they appear to be distinct structures that are differentially regulated.

We recently identified a new regulator of FA disassembly termed ZF21 that promotes cell migration [14]. ZF21 is a member of a protein family that shares the FYVE domain for binding phosphatidylinositol-3-phosphate in the plasma membrane and vesicles. Unique domains of ZF21 bind several cytoplasmic proteins reported to play roles in FA disassembly [15], [16]. These include calpain, which cleaves FA structural proteins [17], FAK, which plays central roles in FA assembly and disassembly [18], SHP-2, which dephosphorylates pY^397^-FAK [19], [20], and tubulin [21], [22]. Since microtubules (MTs) are essential for the regulation of FA disassembly by ZF21 [14], it is most likely that ZF21 binds to vesicles moving along with the MTs and conveys the associated factors to the FAs for disassembly of the later. Although most ZF21 associates with intracellular vesicles, a fraction of the protein has indeed been observed at FAs, presumably localizing there via direct interaction with FAK [14]. Since both FAs and invadopodia play roles in cell invasion, it is possible that ZF21 affects the structure and function of invadopodia directly or indirectly. In the present study, we demonstrate that ZF21 promotes cell migration by simultaneously destabilizing FAs and promoting ECM degradation at the invadopodia. Thus, ZF21 appears to play multiple key roles to promote cancer invasion.

## Materials and Methods

### 2.1. Cells, Antibodies, Plasmid and Reagents

HT1080 and MDA-MB231 cells were obtained from the American Type Culture Collection (Manassas, VA). Cells were cultured in DMEM (Invitrogen), supplemented with 10% fetal bovine serum, penicillin, and streptomycin (Invitrogen Corp.). All cells were cultured at 37°C under a 5% CO_2_, 95% air atmosphere. A polyclonal anti-ZF21 antibody was prepared as described previously [14]. We used commercially available antibodies to detect actin (C4, Millipore) and Tyr397-phosphorylated FAK (BIOSOURCE). Rhodamine-Phalloidin was purchased from Invitrogen. MMI270 (a synthetic hydroxamic MMP inhibitor, a kind gift from Novartis Pharma AG, Basel, Switzerland) and Nocodazole (Sigma) were used at 10 µM and 5 µM, respectively. All other chemical reagents were purchased from Sigma or Wako, unless otherwise indicated.

### 2.2. Knockdown experiments using shRNA

The coding sequence of the shRNA used to knockdown human ZF21 expression is as follows: shZF21#1; 5′-caccgcagtgtgacgccaagtttgacgaatcaaacttggcgtcacactgc-3′, shZF21#2; 5′-caccgcgtaccacagggattaatcccgaaggattaatccctgtggtacgc-3′. All shRNA-expressing lentiviral vectors were generated and used according to the manufacturer's instructions (Invitrogen).

### 2.3. Cell migration and Matrigel invasion assays

The transwell migration and matrigel invasion assays were performed as described previously [14]. Briefly, transwells with 8-μm pore size filters (Corning) pre-coated on both sides with fibronectin or covered with matrigel (BD Biosciences) were inserted into 24-well plates. DMEM containing 10% FBS was added to the lower chamber and a cell suspension (5×10^4^ cells) was placed in the upper chamber. The plates were incubated at 37°C in a 5% CO_2_ atmosphere for 6 hr. After incubation, the cells that had migrated to the lower side were stained with 0.5% crystal violet solution or Giemsa solution and counted using a light microscope at ×200 magnification. Values represent averages from 5 fields.

### 2.4. Fluorescent Gelatin Degradation Assay

The Fluorescent Gelatin Degradation Assay was performed as described previously [23]. Oregon Green-labeled gelatin was obtained from Invitrogen. 4-well glass slides (Thermo Fisher Scientific) were coated with 50 μg/ml poly-l-lysine for 20 min at room temperature, washed with PBS, and fixed with 0.5% glutaraldehyde for 15 min. After 3 washes, 0.2% fluorescently labeled gelatin in PBS was incubated for 10 min at room temperature. After washing with PBS, coverslips were incubated in 5 mg/ml sodium borohydride for 5 min and washed 3 times in PBS. To assess the ability of cells to form invadopodia and degrade the gelatin, cells were plated on Oregon Green-coated coverslips in complete medium with or without EGF (10 ng/mL) and incubated at 37°C for 3 hr to 9 hr.

### 2.5. Immunofluorescence Microscopy

Cells were fixed with 4% paraformaldehyde and permeabilized using 0.1% Triton-X100 in PBS for 12 min. After the cells were blocked in PBS containing 5% goat serum and 3% bovine serum albumin, they were incubated with primary antibodies. All primary antibodies were visualized with an Alexa 488-conjugated goat anti-mouse antibody (Invitrogen). Cells for analysis of gelatin degradation assay were stained for F-actin using Rhodamine phalloidin (Invitrogen). Images of cells were captured with Leica ASMDW with CCD camera (Leica) or IX81/Fluorview1000 (Olympus).

### 2.6. Statistical Analysis

Data represent the means ± S.D or S.E.M. The unpaired Student's t test was used for analyzing differences between experimental groups.

## Results

### 3.1. ZF21 promotes cancer cell invasion

**Figure 1:**
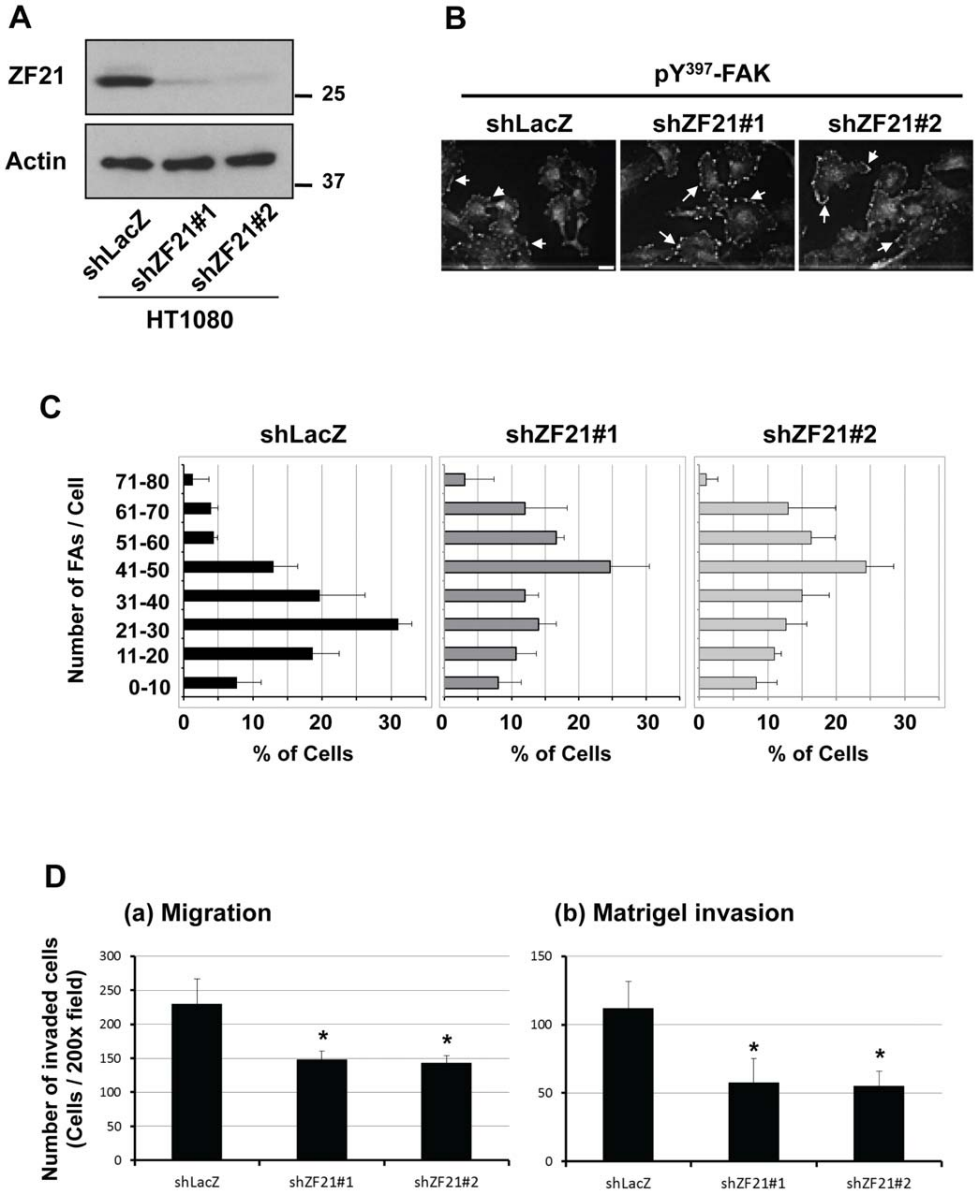
ZF21-knockdown increases the number of FAs and reduces the invasive activity of HT1080 cells. **A.** Expression of ZF21 in HT1080 cells was knocked down using either of two shRNA sequences targeting ZF21 mRNA (shZF21#1 and #2). Endogenous ZF21 was detected by Western blot analysis by using a polyclonal anti-ZF21 antibody. **B.** The cells expressing shLacZ (top) or shZF21#1 and #2 (middle and bottom) were seeded onto glass coverslips. After 48 h, FAK phosphorylated at Tyr^397^ (pY^397^-FAK) was visualized with a specific antibody. Scale bar, 10 µm. **C.** Quantitative analysis of the number of pY^397^-FAK positive punctate signals. The number of pY^397^-FAK positive punctate singals was counted in 100 cells. The experiment was independently repeated three times. **D.** The cells expressing shLacZ or shZF21#1 and #2 were subjected to a migration (a) and a matrigel invasion assay (b) using a transwell chamber equipped with filters coated with fibronectin or matrigel. As an attractant, fetal bovine serum was added in lower chamber. Error bars indicate the means±S.D. (n = 3).*, p<0.05 (Student's t test).

To evaluate the effects of ZF21 on cancer cell invasion, we used human sarcoma HT1080 cells which have been shown to exhibit MMP-dependent invasion into reconstituted basement membrane “matrigel”. We first determined whether ZF21 regulates FA dynamics in monolayer cultures of HT1080 cells. ZF21 is constitutively expressed in HT1080 cells and lentivirus-mediated transduction of either of two shRNA sequences targeting ZF21 mRNA (shZF21#1 and shZF21#2) decreased the level of ZF21 protein (Fig. 1A). Immunohistochemistry using an antibody against Tyr^397^-phosphorylated FAK visualized FAs as small spots localizing at the periphery of cells (Fig. 1B, shLacZ), which were increased in number in the knockdown cells (Fig. 1B, shZF21#1 & shZF21#2). The number of FAs was counted (Fig. 1C) and the results indicate that ZF21 plays a role in promoting turnover of FAs in monolayers of HT1080 cells, similar to what we observed in a previous study using MDA-MB231 cells [14].

We next evaluated cell migration using the transwell chamber assay and we observed that the knockdown cells showed reduced migration compared with the control cells (Fig. 1D–a). The knockdown cells also showed reduced invasion into matrigel compared with the control cells (Fig. 1D–b). Interestingly, ZF21 knockdown had a greater effect upon invasion (shZF21#1: 49%, shZF21#2: 51%) than upon migration (shZF21#1: 36%, shZF21#2: 38%), suggesting that the effect of ZF21 upon cell invasion may not be a simple reflection of its effect upon cell migration.

### 3.2. ZF21 regulates tumor cell invasion in a microtubule-dependent manner

**Figure 2:**
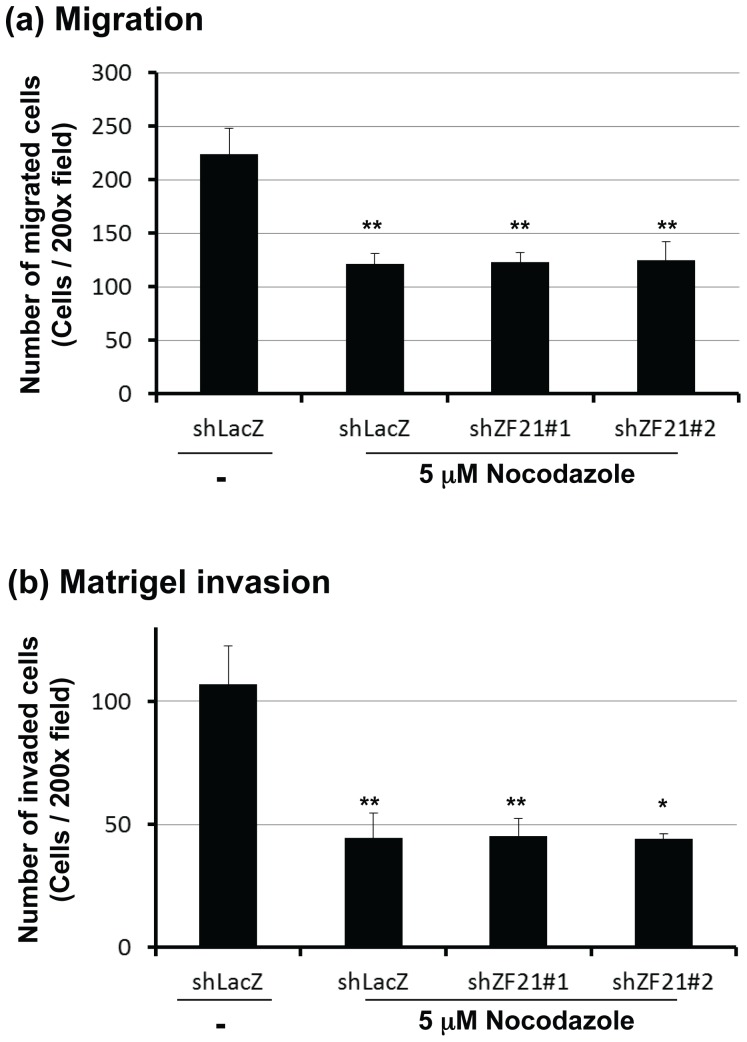
Microtubule disruption diminishes the effect of ZF21-knockdown on the invasive activity of the cells. In the presence of 5 µM nocodazole, the same set of cells indicated in Fig. 1D was subjected to migration (a) and matrigel invasion assays (b). Error bars indicate the means±S.D. (n = 3).*, p<0.05 **, p<0.01 (Student's t test).

In a previous study, we demonstrated that microtubules (MTs) are necessary for regulation of FA disassembly by ZF21 [14]. Therefore, we next tested whether MTs are required for the regulation of cellular migration and invasion by ZF21 using nocodazole, an inhibitor of tubulin polymerization. Nocodazole inhibited both migration (Fig. 2a) and invasion (Fig. 2b) of the control cells (shLacZ) to 42 and 54%, respectively. Furthermore, the knockdown of ZF21 was negligible in the nocodazole-treated cells. Nocodazole treatment decreased migration in shLacZ, shZF21#1, and shZF21#2 by 54%, 55%, and 56% and invasion by 42%, 42%, and 41%, respectively. Since MTs are important for secretion of proteins, it is plausible that ZF21 regulates migration and invasion of the cells through MT-dependent secretion of proteins.

### 3.3. ZF21 regulates ECM degradation

**Figure 3:**
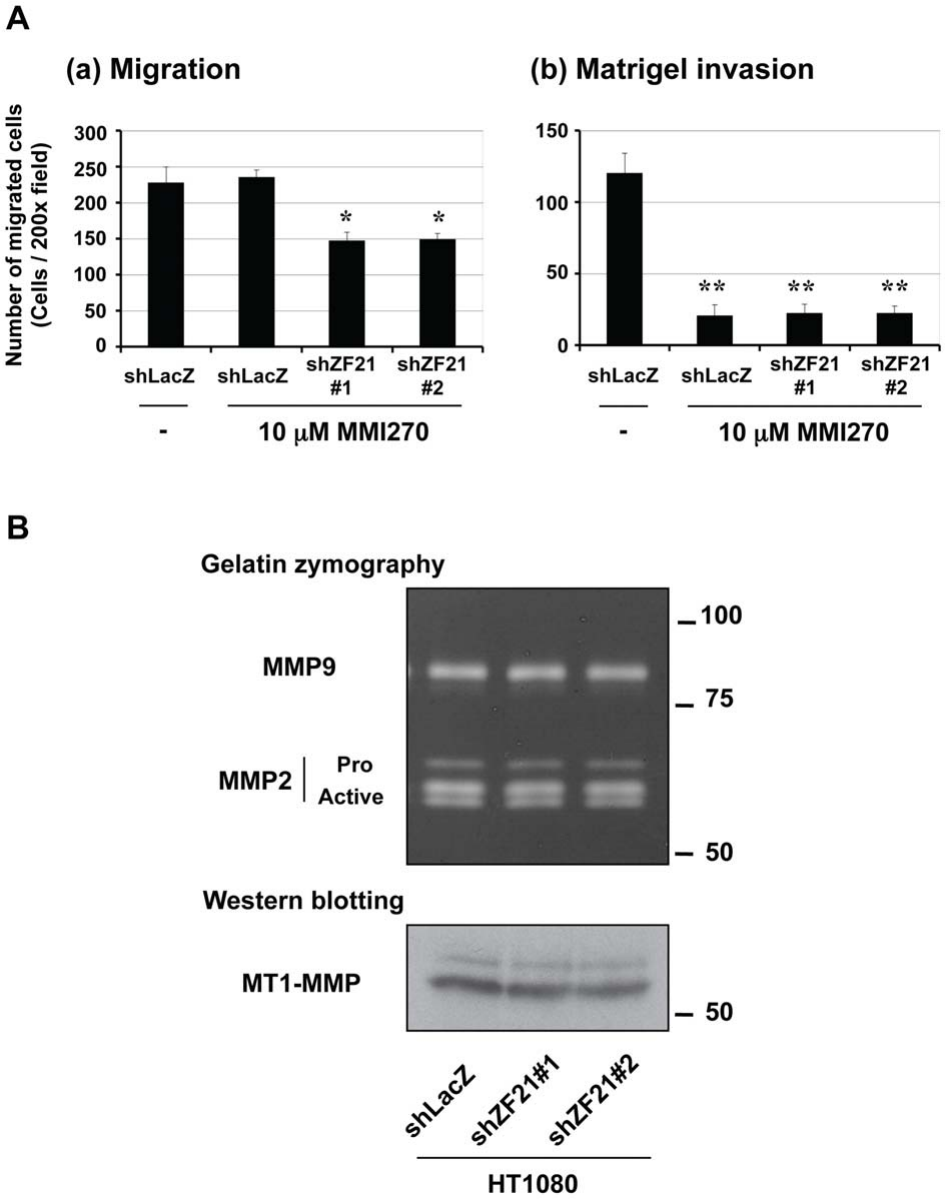
ZF21-mediated matrigel invasion was suppressed by MMP inhibition. **A.** In the presence of 10 µM MMI270, the same set of cells indicated in Fig. 1D was subjected to migration (a) and matrigel invasion assays (b). Error bars indicate the means±S.D. (n = 3).*, p<0.01 **, p<0.005 (Student's t test). **B.** The cultured media of HT1080 cells expressing shLacZ or shZF21#1 and #2 were subjected to gelatin zymography analysis to detect MMP-2 and MMP-9. The same samples indicated in Fig. 1A were subjected to the Western blot analysis to detect MT1-MMP using a specific antibody. The expression levels of MT1-MMP in the cells were normalized by those of actin indicated in Fig. 1A.

MMPs are important players for cancer cell migration and invasion by degrading the ECM [24]. Therefore, we addressed the question whether MMPs play a role in the ZF21-dependent migration and invasion by treating the cells with a synthetic MMP inhibitor MMI270. MMI270 treatment did not alter the migration of the control cells (shLacZ) (Fig. 3A–a). While the ZF21 knockdown cells exhibited less migratory activities, it was not significant and was same as observed in the absence of MMI270 (Fig. 1D–a).

However, MMI270 treatment strongly inhibited invasion of the control cells (Fig. 3A–b). In Fig. 1D–b, we observed that depletion of ZF21 decreased invasion activity to around 50% of the control cells and upon MMPI270 treatment this effect was further enhanced hence, showing that the invasion is almost entirely dependent on the MMP activity of the cells.

Since ZF21 affected invasion of the cells (Fig. 1D–b), ZF21 may employ MMP activity of the cell and overall regulate the invasion. Therefore, we analyzed the effect of ZF21 knockdown on both expression and secretion of three major MMPs that play crucial roles during matrigel invasion by HT1080 cells [25]. As shown in Fig. 3B, ZF21 knockdown had no effect on the expression of MT1-MMP, MMP-2 or MMP-9. Furthermore, ZF21 knockdown did not affect the activation of MMP-2 mediated by the proteolytic activity of MT1-MMP (Fig. 3B). Thus, the decrease in cell invasion resulting from knockdown of ZF21 expression is not due to decreased expression or secretion of the MMPs.

### 3.4. ZF21 regulates degradation of the ECM at invadopodia

The regulation of MMP activity including that of MT1-MMP within particular regions of the cell surface such as invadopodia and the invasion edge is not fully understood and might differ between these regions. For example, MT1-MMP is reported to be recruited to invadopodia via a recycling pathway rather than by direct transport from the Golgi [26], [27]. In addition, MT1-MMP activity might be modulated by cytoplasmic proteins that are recruited to the invadopodia and interact with the cytoplasmic tail of MT1-MMP. Since ZF21 may affect these processes, we examined whether depletion of ZF21 affects degradation of the ECM at invadopodia. Invadopodia can be observed as discrete actin-rich spots located at the cell-ECM interface and they are more easy to discern that the invasive edge of cells. MDA-MB231 cells are a good model because the invadopodia are quite pronounced and stable. Therefore, we employed MDA-MB231 cells to study the effect of ZF21 on the cellular invasion machinery. The role of ZF21 on FA disassembly in MDA-MB231 cells has been reported previously [14], [28].

**Figure 4:**
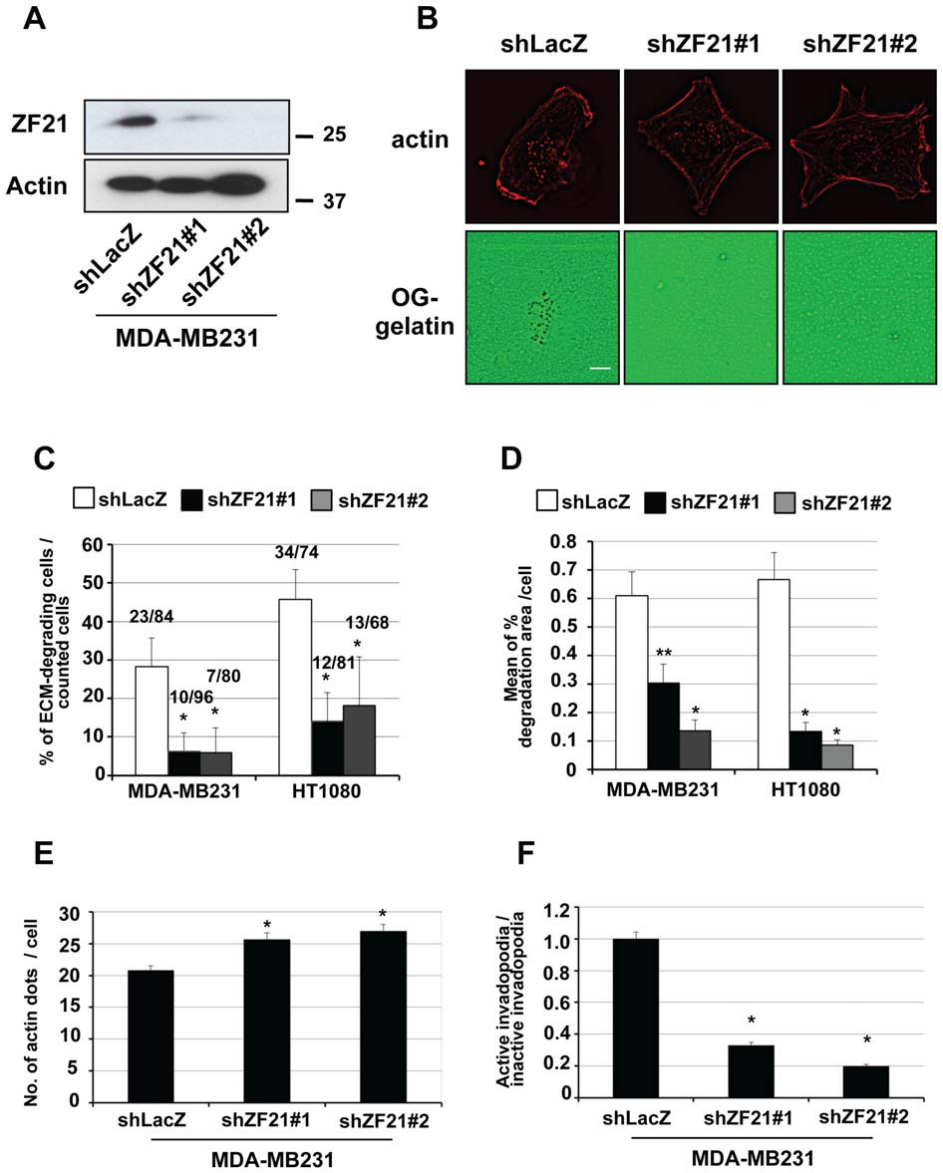
ZF21 knockdown reduces invadopodia-mediated ECM degradation. **A.** Expression of ZF21 in MDA-MB231 cells was knocked down using either of two shRNA sequences targeting ZF21 mRNA (shZF21#1 and #2). Endogenous ZF21 was detected by Western blot analysis by using a polyclonal anti-ZF21 antibody. **B.** The cells expressing shLacZ (top) or shZF21#1 and #2 (middle and bottom) cultured on Oregon Green-labeled gelatin-coated glass coverslips. After fixation, the cells were stained with phalloidin for localization of F-actin (red on left panels). Degraded ECM appears as dark areas (reduced green fluorescence on right panels). (scale bar, 10 μm). **C.** Percentage of cells exhibiting ECM degradation. Number of cells stained with phalloidin was counted and merged with the ECM degraded area. The averaged number of the ECM-degrading cells and total counted cells are indicated upon each column in graphs (ECM-degrading cells/total counted cells). Ratio of the ECM-degrading cells is represent as the means ± S.D (n = 3), *, P<0.0001, **, P<0.005. **D.** Average degradation area per cell is calculated and presented. Error bars indicate the means ± S.E.M. *, P<0.0001, **, P<0.005. **E.** Number of punctate actin signals was counted for 450–500 cells. Average number of actin dots per cell is presented. Error bars indicate the means ± S.E.M *, P<0.0005. The experiment was repeated six times. **F.** Ratio of ECM-degrading actin dots (active invadopodia) to no ECM-degrading actin dots (inactive invadopodia) is presented. Error bars indicate the means ± S.E.M *, P<0.0005.

The expression of ZF21 in MDA-MB231 was knocked down using the same set of the lentivirus vectors (shZF21#1 and shZF21#2) indicated in Fig. 1A (Fig. 4A). The cells were seeded and cultured on fluorescent Oregon green-labeled gelatin (OG-gelatin) coating a slide glass. Invadopodia were visualized by punctate actin signals in the center of the cells (Fig. 4B, actin). ECM (OG-gelatin) degradation was revealed by areas of reduced Oregon-green fluorescence. The areas of ECM degradation overlapped with the punctate actin signals and therefore represented invadopodia exhibiting ECM-degrading activity (Fig. 4B, OG-gelatin). There was a comparable number of punctate actin signals in cells following knockdown of ZF21, although gelatin degradation was strongly suppressed (Fig. 4B, shLacZ versus shZF21#1 and shZF21#2).

Although HT1080 cells also form invadopodia (data not shown), the greater mobility of these cells makes it difficult to observe the overlap between the punctate actin signals and the areas of ECM degradation. Therefore, the ECM-degrading activity of HT1080 was estimated as the ratio of the ECM-degrading cells (Fig. 4C) or total degradation area per cell (Fig. 4D) and compared with the invadopodia-mediated ECM degradation by MDA-MB231 cells. Knockdown of ZF21 expression effectively decreased the extent of ECM degradation in both types of cell.

The number of the punctate actin signals within the ZF21 knockdown MDA-MB231 cells was similar to that in the control cells (Fig. 4B, actin; shLacZ versus shZF21). In fact, number of actin signals counted in the knockdown cells was increased slightly compared to the control (Fig. 4E). However, the number of actin signals that overlap with the gelatin degradation spots was decreased dramatically (Fig. 4F). Thus, it is clear that ZF21 regulates the ECM-degrading activity of invadopodia without affecting formation of the actin-based invadopodia structures.

### 3.5. ZF21 promotes accumulation of MT1-MMP at invadopodia

**Figure 5:**
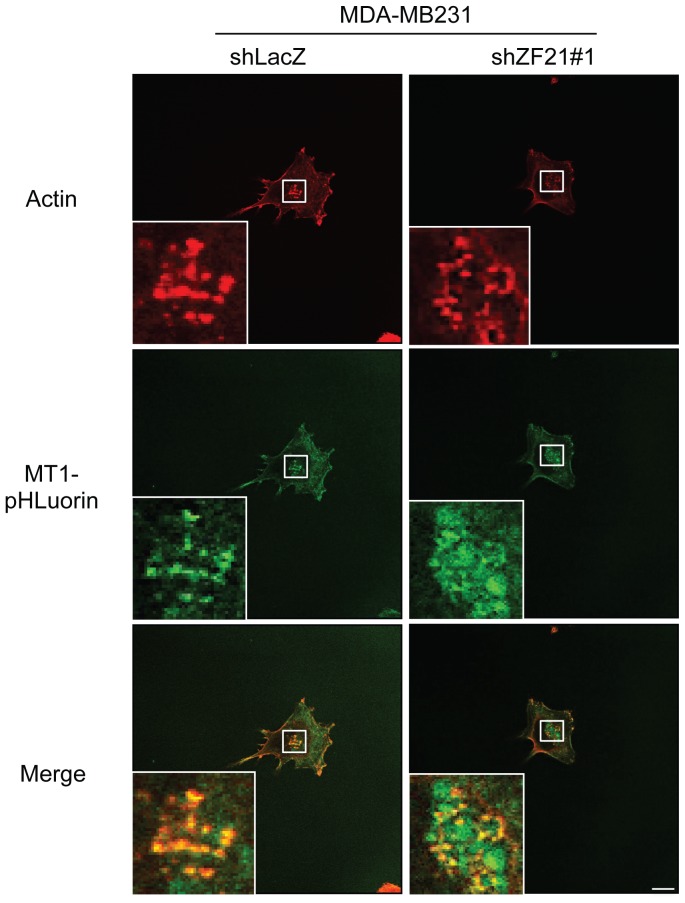
ZF21 regulates localization of MT1-MMP to invadopodia. MT1-MMP-pHLuorin (MT1-pHLuorin) was transiently expressed in the MDA-MB231 cells expressing either shLacZ or shZF21#1. The cells were cultured on cover slips coated with 50 µg/ml fibronectin for 6 hours. After fixation, the cells were stained with Rhodamine-labeled phalloidin to visualize F-actin. The localizations of F-actin (red on top panels) or MT1-MMP-pHLuorin (green on middle panels) were observed using confocal microscopy. The merged pictures of red and green fluorescence were shown on bottom panels. The boxed areas are shown at higher magnification in the panels. (scale bar, 10 μm).

Since MT1-MMP is a major ECM-degrading MMP at invadopodia, we asked whether the knockdown of ZF21 in MDA-MB231 cells altered localization of MT1-MMP at the invadopodia. To monitor if MT1-MMP specifically localizes on the cell surface, we used MT1-MMP tagged with pHLuorin, a pH-sensitive GFP derivative (MT1-pHLuorin) and expressed it in the cells expressing either shLacZ or shZF21#1 (Fig. 4A). Fluorescence of the pHLuorin becomes bright at the neutral pH range but not in the acidic conditions. Therefore, MT1-pHLuorin enables us to monitor dynamic turnover of MT1-MMP on the cell surface as we have previously reported [27]. Both cells expressing shLacZ or shZF21#1 formed invadopodia-like actin-based structures even after expression of MT1-pHLuorin (Fig. 5, actin). We find that MT1-pHLuorin accumulated at the actin puncta in the control cells expressing ZF21 protein (Fig. 5, MT1-pHLuorin, shLacZ). However, depletion of ZF21 diminished the localization of MT1-pHLuorin to invadopodia (Fig. 5, MT1-phLuorin, shZF21#1). Thus, ZF21 regulates localization of MT1-MMP at the actin-based invadopodia structures.

## Discussion

Invadopodia and FAs are two characteristic cellular structures interacting with the ECM. These structures are visible when cells are cultured atop the ECM. Although both structures coexist within the cell, a reduction in the number of FAs is associated with increased mobility and an increase in the number of invadopodia exhibiting ECM-degrading activity is associated with greater invasiveness. MMP activity is essential for HT1080 cell invasion but not cell migration (Fig. 3). In a previous study, we found that ZF21 regulates disassembly of FAs [14]. ZF21 binds to phosphoinositides and associates with transport vesicles [14]. ZF21 associated with vesicles can bind cytoplasmic proteins required for FA disassembly such as calpain, SHP-2, tubulin, and FAK [14], [15]. Therefore, it is plausible that ZF21 transports these disassembly factors to FAs via MT-associated vesicles. Thus, ZF21 is thought to bind and convey particular cytoplasmic proteins to FAs by associating with transport vesicles. ZF21 may also convey factors that regulate ECM degradation at the invadopodia via a mechanism analogous to its role in the regulation of FA turnover.

In spite of the evidence indicating that ZF21 regulates the ECM degradation activity associated with invadopodia, the number of invadopodia as assessed by actin visualization was unchanged following depletion of ZF21. In addition, knockdown of ZF21 expression did not affect the overall secretion of MMP-2, -9 or MT1-MMP at the cell surface. The ability of MT1-MMP to activate MMP-2 was also unaffected by knockdown of ZF21.

The depletion of ZF21, however, reduces the accumulation of MT1-MMP to the actin-based invadopodia structures as demonstrated in Fig. 5. ZF21 was originally identified as an interacting protein for the cytoplasmic tail (CPT) of MT1-MMP by the yeast two-hybrid analysis, although direct interaction between the two proteins was not observed. ZF21 may transport certain cytoplasmic proteins via vesicles to the invadopodia and that these proteins may regulate MT1-MMP activity locally. The CPT of MT1-MMP can be posttranslationally modified via phosphorylation [29], [30] and palmitoylation [31]. The CPT also binds several proteins, such as caveolin-1 [32], AP2 adaptor protein mediating internalization [33], ADI1/MTCBP-1 [34], p27RF-Rho [23], FIH-1 [35], gC1qR [36], and possibly also unidentified PDZ binding proteins because the CPT contains a PDZ motif at its C terminus [37]. ZF21 may play a role in mediating interaction of the CPT with the above-mentioned factors.

ZF21 does not affect overall secretion of MT1-MMP, MMP-2 or MMP-9, but nevertheless may regulate transport of these proteins to a particular region of the membrane. Indeed, accumulation of MT1-MMP at invadopodia was abrogated in the MDA-MB231 cells lacking ZF21 expression as demonstrated in Fig. 5. This observation explains why ZF21 depleted cells exhibits reduced ECM-degrading activity specifically at the invadopodia. However, this does not exclude the possibility that ZF21 may also affect other proteases at the invadopodia. In a previous study, we examined transport of MT1-MMP to invadopodia using the techniques of fluorescence recovery after photobleaching (FRAP) and fluorescence loss in photobleaching (FLIP) [27]. In these studies, we employed inhibitors of vesicle transport such as breferdin, bafilomysin, and dynaso, and reached the conclusion that MT1-MMP is not transported directly from the Golgi to invadopodia, but rather via the endosome/lysosomal recycling pathway. Thus, it is possible that ZF21 affects preferentially the lysosomal transport pathway rather than the default pathway from the Golgi to the surface, depending on the functions of the proteins associated with transport vesicles via ZF21. In addition to the targeted transport of MT1-MMP and MMPs to invadopodia, internalization of MT1-MMP at the invadopodia may act as a rate-limiting factor for ECM degradation, since a recent computational model-based simulation of invadopodia-mediated ECM degradation predicted that rapid turnover of MT1-MMP at the invadopodia is necessary for effective ECM degradation [27].

Computational analysis of networks of proteins associating with FAs and invadopodia identified PI3K and PKCα as hub molecules regulating the balance between formation of FAs and invadopodia. Analysis of head and neck cancer clinical specimens revealed that cells with high PI3K coupled with low PKCα activity exhibited increased invasiveness [38]. Interestingly, several reports indicate that the phosphatidylinositol 3-phosphate (PtdIns3P) produced by PI3K such as PI3K-C2β or PIK3C3 have a crucial role as an intracellular second messenger in the signaling pathway regulating multiple cellular behaviors including cell migration [39], [40]. The balance of PI3K/PKCα activation may be important for the production of PtdIns3P. On the other hand, the binding of ZF21 to PtdIns3P may change the phosphorylation status or intracellular localization of the phospholipid, and thereby affect the signaling pathway regulating the FAs and invadopodia. This is an interesting possibility that remains to be tested in future.

In conclusion, ZF21 not only regulates cell migration via its effect upon FA stability, but also regulates invasion via its effect upon ECM-degradation activity at invadopodia. Although the exact mechanism is unclear, ZF21 appears to regulate both FAs and cell invasion in a coordinated manner. Development of inhibitors targeting ZF21 activity may contribute to cancer therapy.
